# Spatiotemporal clustering of suicide attempt in Kermanshah, West-Iran

**DOI:** 10.3389/fpsyt.2023.1174071

**Published:** 2023-07-31

**Authors:** Alireza Zangeneh, Nahid Khademi, Naser Farahmandmoghadam, Arash Ziapour, Reyhane Naderlou, Somayyeh Shalchi Oghli, Raziyeh Teimouri, Komali Yenneti, Shahrzad Moghadam

**Affiliations:** ^1^Social Development and Health Promotion Research Center, Health Institute, Kermanshah University of Medical Sciences, Kermanshah, Iran; ^2^Medical Ethics and History of Medicine Research Center, Tehran University of Medical Sciences, Tehran, Iran; ^3^Clinical Research Development Center, Imam Khomeini and Mohammad Kermanshahi Hospitals, Kermanshah University of Medical Sciences, Kermanshah, Iran; ^4^Cardiovascular Research Center, Health Institute, Imam-Ali Hospital, Kermanshah University of Medical Sciences, Kermanshah, Iran; ^5^Geography and Urban Planning, University of Zanjan, Zanjan, Iran; ^6^Department of Health Education and Promotion, School of Public Health, Tehran University of Medical Sciences, Tehran, Iran; ^7^UniSA Creative, University of South Australia, Adelaide, SA, Australia; ^8^School of Architecture and the Built Environment, Faculty of Science and Engineering, University of Wolverhampton, Wolverhampton, United Kingdom

**Keywords:** suicide attempts, spatial pattern of suicide, spatial analysis, geographic information system, Kermanshah, Iran

## Abstract

**Background:**

A suicide attempt is a major societal problem because it imposes high costs on societies worldwide. This paper analyses the spatiotemporal clustering of suicide attempt in Kermanshah, Iran from 2006–14.

**Methods:**

This study draws on 18,333 individuals (7,234 males and 11,097 females) who attempted suicide across the Kermanshah province. Data was collected from the records of individuals registered in hospitals across the Kermanshah province between 2006 and 2014. Mean Center, Standard Deviational Ellipse (SDE), Moran’s I and Kernel Density Estimation (KDE) in Arc/GIS10.6 software were used for the analysis of the spatial distribution of suicide attempt, while the chi-squared test in SPSS was used to examine the different demographic variables between groups within/outside spatial clusters of suicide.

**Results:**

The results show that a total of 18,331 suicide attempts (39.46% male and 60.53% female) were reported between 2006 and 2014 in the Kermanshah province. The spatial pattern of suicide attempts was clustered in 16 clusters (6 high clusters and 10 low clusters) and statistically significant differences were found within and outside the hotspots of suicide attempts. Most hot spots were formed in and around cities. Younger people were at a greater risk. The rate of suicide attempts reduced in illiterate people and increased in people with university degrees. Unmarried people were associated with a higher risk of suicide attempt than was married status for both males and females.

**Conclusion:**

The results of this study could help public health practitioners and policymakers in Iran prioritize resources and target efforts for suicide attempt prevention.

## Introduction

Suicide attempts is a serious public health matter and is a leading cause of death across the world (1). Yearly, more than 703, 000 people attempt suicide. In 2019, suicide was the fourth leading cause of death in people aged 15–29, while more than 77% of suicides occurred in low- and middle-income countries (1, 2). Additionally, the number of suicide deaths among males in richer countries is three times that of females, while 1.5 males die by suicide per a woman in low- and middle-income countries (1).

In most countries, a suicide has many personal, family, and social impacts. For example, suicide attempts have strong emotional impact on not only the deceased’s friends and families, but also on the survivors themselves. It may include the medical costs and lost income for families, as well as the loss of employer’s productivity (3–5). In general, suicides impose huge social and economic costs to people, families, and society. In addition, suicide attempts have been a ripple effect that has had a major impact on our lives.

Suicide attempts are preventive. Many actions may be done to prevent suicide attempts at individual, community and national levels. Existing scholarship indicates that most people who attempt suicide have characteristics such as anxiety, aggression, and withdrawing from social interactions (6). Social withdrawal reflects individuals’ interactions and feelings of connectedness with the environment surrounding them which it has been linked to suicidal thoughts and/or behaviors (7). Major referrals to emergency departments in hospitals that are all voluntary, harmed themselves by different methods and motives (3, 8). However, suicidal behaviors are also influenced by different risk factors such as age, gender, ethnicity and socioeconomic conditions (9–12). For example, between 1991 and 2017 in the United States, black teenagers were the only population group with an increase in the rate of suicide attempts (12). Female teenagers have more non-fatal suicidal attempts than males (13), while teenagers from lower socio-economic backgrounds are at higher risk of suicide (14). In Canada, 10% of suicide related deaths occurred in the age group of 10 to 14 years, while 23% of deaths occurred in adolescents aged 15 to 19 (15). In New Zealand, the suicide attempts of children and adolescents from lower socioeconomic class are determined to be 31 times more likely than those of children and adolescents from higher socioeconomic status (16). In Asia, relationship issues, environmental problems and academic performance are common factors for suicide attempts in young people. In Singapore, most suicide attempts occur among teenagers and young people aged 15–24 (17). Self-harming behaviors like suicide amongst teenagers in Hong Kong (23.5%) were less compared with their peers in the United States (32%). The low rate of self-harm among young people in Hong Kong may be related to the differences between the Eastern and Western cultures, with strong family structures in Asian cultures (18). Suicide rate is also higher in Korea among the elderly and those over 65 years of age and is affected by economic conditions (19). Besides, marital status was related to various suicidal risk levels. All types of changes in marital status (widowed, divorced, married) are considered risk factors for suicidal attempts and suicidal behavior (20). The risk of suicide for divorced and widowed people depends on age: older people are at higher risk.

Research also shows high correlation between suicide attempts and residential environment and economic conditions (18, 21), but there is a little literature on the relation between suicides and community welfare. It is generally argued that the suicide rate is affected by the degree of urbanization, with the suicide the rate in rural areas being higher than that in urban areas (22, 23). In particular, the suicide rate among males in rural areas is higher than that of females (21, 24). However, the rate of suicide attempts in urban and rural areas has changed considerably in recent times (19), while most scholarship has been limited to just a few areas (23, 25). Existing literature also indicates a possibly very low relation between the specification of rural and urban areas and suicide rates at the national level (4, 26, 27).

Accident datasets reported in shape of tables or graphs are too involved to realize comprehensively (28). Indeed, it is essential to create an impressive spatial–temporal analyst methodology to explore the big accident datasets (29). Clustering is one of the most noteworthy unsupervised learning algorithms which has been widely used in determining hotspots (30). Wherein, events are grouped according to their strong similarity. There are four major types of clustering algorithms such as partitional, hierarchical, grid-based, and density-based clustering algorithms (31). Partitional algorithms contain k-means and k-medoids. This methodology requires predefined cluster numbers. Therefore, it is not used in many applicants. Hierarchical algorithms contain clustering using delegate and balanced iterative reducing and clustering using hierarchies, being effectual for summarizing and imagining data. Nevertheless, this methodology is difficult to scale up since each indecision needs to assess many events. Grid-based algorithms contain statistical information grid and clustering in quest. Partitioning and hierarchical algorithms are appropriate to recognize spherical-shaped clusters. Density-based algorithms contain Density-Based Spatial Clustering of Applications with Noise (DBSCAN), ordering points to recognize the clustering structure, and DBSCAN, being popular techniques for recognizing clusters of ideal shape. In density-based spatial clustering of applications with noise and ordering points to recognize the clustering structure, density is calculated by recounting the number of events in a surrounding area defined by a bandwidth. This is highly sensible to the bandwidth valuation applied. To deal with it, DBSCAN or KDE can be used, which is a non-parametric density estimator effective technique. The KDE methodology could efficiently reduce the effectiveness of noise by equally allocating noise into the input data (31, 32).

GIS algorithm, KDE could build a density scheme, which indicates the density of the points (33). DBSCAN, meanwhile, only recreates the form of the clusters and does not mention the density of the clusters. The density differentiation among clusters are not highlighted (34). Moreover, DBSCAN could not group well events with interchanged densities is the biggest drawback of this methodology (35). The great advantage of DBSCAN of applications is that it can identify clusters with arbitrary shapes (28). Moreover, differ from several clustering methodologies, KDE and DBSCAN do not require predefined cluster numbers (36).

Furthermore, suicide attempts also differ widely across geographical areas. Previous researches exploring differences in rural and urban suicide attempts rates observed higher rates of suicide attempts in rural regions, with rural–urban disparities increased (4, 37). Researches using spatial analysis techniques have been able to identify geographical area with high suicide attempts rates relative to other areas, also highlight region characteristics associated with elevated suicide attempts rates within specific geographic areas (4). A suicide attempts cluster might be defined as a higher number of suicides attempts occurring within a defined space and/or time than would otherwise be expected for a specific area (4). Other study revealed the spatial patterning of suicide attempts in Taiwan and found that the factors most strongly associated with region suicide attempts rates were median household income, population density, and single parent households (38). Another study in Quebec revealed that clusters of heightened suicide attempts risk were most likely to be showed in distant rural regions and in neighborhoods with higher proportions of immigrations and individuals living alone (39).

Notwithstanding, despite the burgeoning literature on suicide attempts across the world there is little scholarship that looks at both sex and age as potential factors affecting suicide rates (19) and cross-cultural perspectives of suicidal behavior, intentional self-harm, and non-suicidal self-injury of young people in a systematic way, especially in the developing world (40). Most of the literature on suicidal behaviors has been focused on Western and Asian developed countries (4, 15, 41, 42). Similarly, studies from Iran shows that the increase in suicide rate in the country is due to unfavorable economic conditions, such as unemployment, poverty and diseases (43–45). Although religion plays a vital role in mollifying suicidal attempts, it may dictate privacy and exacerbate the stigma of mental health and suicide among Arab societies (46).

As previous studies declaration, doing more research on suicide as an important social issue is required in developing countries (4). This research examined spatial patterns of suicide attempts over 8 years in an Iranian province. In this context, this study focuses on exploring the spatiotemporal clustering of suicide attempt in Kermanshah, Iran for the period of 2006–2014. Understanding the spatial distribution of suicide attempts can play an important role in predicting family and community socioeconomic situation and individual characteristics (4), and help in proper planning for prevention of suicide attempts (4). Following this introduction, the rest of the paper follows the structure: section 2 briefly describes the case study and outlines the research methodology adopted in this study. Section 3 presents the results and findings of this research. Finally, the paper ends with some discussion and conclusions.

## Materials and methods

### Study area

The Kermanshah with an area of 24,998 square kilometers located in a Zagros Mountain range west of Iran at latitude 34°19′N, longitude 47°7′E. According to the Census of Population and Housing in 2016, the population of Kermanshah was 1,952,434. This province consists of 14 townships, 30 districts, 29 cities, 85 hamlets, and 3,163 villages, and has 69.66% urban, and 30.15% rural population (47). Kermanshah has been facing several socioeconomic challenges including increased unemployment, poverty, cancer, and suicide, and inequality accessibility to healthcare centres among urban and rural areas (47, 48).

### Study population

This study was conducted on 18,333 individuals (7,234 males and 11,097 females) attempted suicide in Kermanshah province, Iran. Data was collected from the records of individuals registered in hospitals across the Kermanshah province between 2006 and 2014. Furthermore, information about people who attempted suicide was collected through forms provided to by the Ministry of Health and completed by health personnel. The documents with information about the patient’s history were checked by medical records experts and sent to the Vice Chancellor of Kermanshah University of Medical Sciences. Information was also collected and archived confidentially. This study was approved by the ethics committee and institutional review board of the Medical Association of the Kermanshah University of Medical Science.

The patients’ records were systematically reviewed. The location data of all people who had attempted a suicide multiple times was recorded only once. Demographic data, including gender, age, marital status, and level of education (illiterate, primary education, the secondary education includes 7-9th grade, and high school includes 10-12th grade, University), was gathered and related to suicide attempts.

### Methods

**Figure 1:**
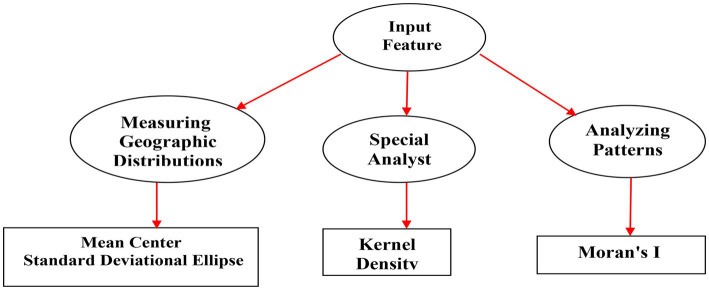
Schematic diagram of the workflow of the paper.

Spatial statistics refer to the use of statistical methodologies to analyze and describe the distributor of events, with a special emphasis on spatial data (49). Presently, there being considerable advancements in both technological and theoretic aspects of GIS science, which have greatly helped spatial statistics. Nevertheless, with the increasing use of spatial statistics in various fields, it plays an essential role in the interaction between methods, computational capabilities, and the distinct characterizations of each discipline (50). This study was pursued the procedures outlined in Figure 1.

Participants’ data were collected from all hospital centers under the supervision of Kermanshah University of Medical Sciences and digitized using GIS with the collected information we run an Arc/Catalog environment. The tests used in GIS included: Mean Center, SDE, Moran’s I and KDE to identify spatial clusters Figure 1.

Mean Center was used for tracking changes in the distribution or for comparing the distributions of different types of suicide attempts. SDE was used to determine whether the distribution of suicide attempt has changed over time. The Moran’s I tool measures spatial autocorrelation of based on both feature locations and feature values simultaneously. Given a set of features and an associated attribute, it evaluates whether the pattern expressed is clustered, dispersed, or random. The tool calculates the Moran’s I Index value and both a z-score and value of p to evaluate the significance of that index. *p*-values are numerical approximations of the area under the curve for a known distribution, limited by the test statistic. The KDE tool calculated the density of suicide attempt in a neighborhood around those features.

In addition, the T-independent test was used to examine the different demographic variables between groups within and Outside Spatial Clusters of Suicide. The findings were examined at confidence level of 95% (*p* ≥ 0.05). Also, all data were analysed by the SPSS software package.

In this study, a suicide cluster is specified as an unusually high number of suicidal attempts occurring closer together in time and/or space than would be expected by odds (51). The Chi-square test was used to examine the correlations between the independent variables and dependent variable. The chi-square goodness of fit test was used to test whether there were statistical differences among different groups. The chi-squared test was used to examine the different demographic variables between groups within and outside spatial clusters of suicide attempts. The findings were examined at confidence level of 95% (*p* ≥ 0.05).

## Results

The results show that there were 18, 331 suicide attempts, of which 39.46% were male and 60.53% were female, during the study period (2006–2014) in Kermanshah province. In terms of education level, most people who attempted suicide received secondary (29.08%) and high school (19.03%) education. The rate of suicide attempts in the illiterate decreased, while it increased in those with higher education (Table 1). Furthermore, 16,763 cases (91.48) were fatal. 39.46% of decedents were male and 60.53 were female (Table 2).

**Table 1:** Demographic characteristics of suicide attempt in the Kermanshah province during the period of 2006–2014.

Variables years	2006	2007	2008	2009	2010	2011	2012	2013	2014	Missing data	Total
Sex *n* (%)											
Male	899 (41.83)	834 (44.96)	735 (63.53)	455 (34.26)	676 (37.49)	653 (38.25)	1,009 (38.22)	867 (38.40)	1,070 (42.730)	36 (48)	7,234 (39.46)
Female	1,250 (58.17)	1,021 (55.04)	1,277 (63.46)	873 (65.74)	1,127 (62.99)	1,054 (61.75)	1,631 (61.78)	1,391 (61.60)	1,434 (57.270)	39 (52)	11,097 (60.53)
Total	2,149 (100)	1855 (100)	2012 (100)	1,328 (100)	1803 (100)	1707 (100)	2,640 (100)	2,258 (100)	2,504 (100)	75 (100)	18,331 (100)
Age *n* (%)											
<14	53 (2.46)	47 (2.54)	55 (2.73)	25 (1.88)	34 (1.90)	25 (1.46)	41 (1.55)	38 (1.69)	37 (1.48)	3 (4)	358 (2.95)
15–64	2052 (95.48)	1722 (92.83)	1931 (95.97)	1,274 (95.94)	1745 (96.78)	1,647 (96.49)	2,563 (97.09)	2,203 (97.56)	2,424 (96.80)	70 (93.33)	17,631 (96.18)
65	31 (1.44)	31 (1.67)	12 (0.60)	16 (1.20)	12 (0.66)	19 (1.11)	19 (0.72)	17 (0.75)	43 (1.72)	0	200 (1.09)
Missing data	13 (0.62)	55 (2.96)	14 (0.70)	13 (0.98)	12 (0.66)	16 (0.94)	17 (0.64)	0	0	2 (2.67)	142 (0.77)
Total	2,149 (100)	1855 (100)	2012 (100)	1,328 (100)	1803 (100)	1707 (100)	2,640 (100)	2,258 (100)	2,504 (100)	75 (100)	18,331 (100)
Education level *n* (%)											
Illiterate	271 (12.61)	155 (8.36)	207 (10.29)	105 (7.90)	105 (5.82)	96 (5.62)	166 (6.29)	147 (6.51)	182 (7.27)	6 (8)	1,440 (7.86)
Primary education	383 (17.82)	242 (13.05)	291 (14.46)	209 (15.74)	271 (15.03)	238 (13.94)	369 (13.98)	280 (12.40)	320 (12.78)	6 (8)	2,609 (14.23)
Secondary education	1,329 (61.84)	1,013 (54.61)	1,301 (64.66)	871 (65.59)	1,219 (67.61)	1,178 (69.01)	1753 (66.40)	1,611 (71.35)	1736 (69.33)	41 (54.67)	12,052 (65.75)
University	73 (3.40)	93 (5.01)	126 (6.26)	91 (6.85)	140 (7.76)	141 (8.26)	255 (9.66)	218 (9.65)	249 (9.94)	3 (4)	1,389 (7.58)
Missing	93 (4.33)	352 (18.97)	87 (4.32)	52 (3.92)	68 (3.77)	54 (3.16)	97 (3.67)	2 (0.09)	17 (0.68)	19 (25.33)	841 (4.59)
Total	2,149 (100)	1855 (100)	2012 (100)	1,328 (100)	1803 (100)	1707 (100)	2,640 (100)	2,258 (100)	2,504 (100)	75 (100)	18,331 (100)

**Table 2:** Characteristics groups in within and outside of the foci of suicide attempt, Kermanshah Province, 2006–2014.

Variables	Within of the foci			Outside of the foci	Total within/outside of the foci	*p*-value
	High clusters	Low clusters	Total High/Low clusters			
Sex *n* (%)						0.001
Male	3,815 (41.50)	2,786 (38.71)	6,601 (40.28)	633 (32.60)	7,234 (39.46)	
Female	5,377 (58.49)	4,411 (61.29)	9,788 (59.72)	1,309 (67.40)	11,097 (60.53)	
Total	9,192 (100)	7,197 (100)	16,389 (100)	1942 (100)	18,331 (100)	
Marital status *n* (%)						0.038
Single	5,243 (57.03)	3,951 (54.90)	9,194 (56.09)	1,059 (54.72)	10,253 (55.95)	
Marital	3,667 (39.89)	2,992 (41.57)	6,659 (40.63)	813 (41.65)	7,465 (40.73)	
Wife’s death	49 (0.53)	45 (0.62)	94 (0.57)	16 (0.82)	110 (0.60)	
Divorced	162 (1.76)	135 (1.87)	297 (1.81)	38 (1.96)	335 (1.82)	
Widow	7 (0.07)	12 (0.16)	19 (0.11)	8 (0.41)	27 (0.14)	
Missing	64 (0.69)	62 (0.86)	126 (0.76)	8 (0.41)	134 (0.73)	
Total	9,192 (100)	7,197 (100)	16,389 (100)	1942 (100)	18,331 (100)	
Education level *n* (%)						0.001
Illiterate	642 (6.98)	527 (7.32)	1,169 (7.13)	278 (13.85)	1,440 (7.84)	
Primary education	1,105 (12.02)	1,088 (15.11)	2,193 (13.38)	416 (21.40)	2,609 (14.22)	
Secondary education	6,339 (68.97)	4,656 (64.70)	10,995 (67.09)	1,057 (54.44)	12,052 (65.75)	
University	685 (7.45)	573 (7.97)	1,258 (7.68)	131 (6.75)	1,389 (7.58)	
Missing	421 (4.58)	353 (4.90)	774(4.72)	67(3.56)	841 (4.61)	
Total	9,192 (100)	7,197 (100)	16,389 (100)	1942(100)	18,331 (100)	
Suicide attempt resulting *n* (%)						0.001
Failed	205 (2.23)	252 (3.50)	457 (2.79)	145 (7.13)	595 (3.24)	
Committed suicide	8,475 (92.20)	6,587 (91.52)	15,062 (91.90)	1701 (87.90)	16,763 (91.48)	
Missing	512 (5.57)	358 (4.98)	870 (5.30)	96 (4.96)	966 (5.27)	
Total	9,192 (100)	7,197 (100)	16,389 (100)	1942 (100)	18,331 (100)	
Mean age (SD)						0.001
Male	18.31 ± 13.22	17.67 ± 13.81	17.99 ± 13.51	15.87 ± 15.46	25.92 ± 21.24	
Female	18.14 ± 13.53	18.30 ± 13.75	18.22 ± 13.64	17.53 ± 14.65	26.98 ± 20.96	
Total	18.21 ± 13.40	18.06 ± 13.77	18.13 ± 13.58	16.99 ± 14.94	26.62 ± 21.05	

**Figure 2:**
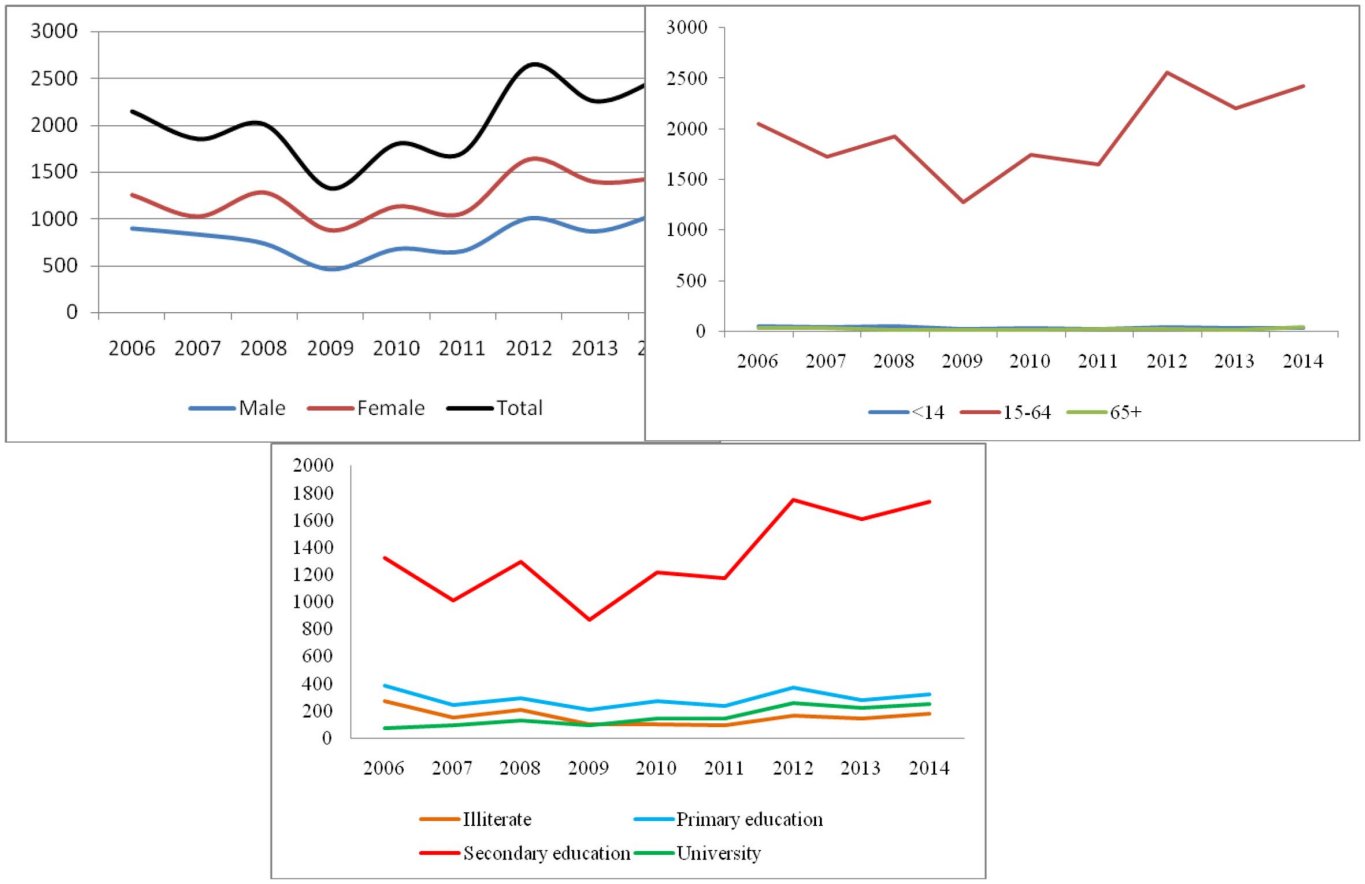
Trend suicide attempt based on gender, age groups, and education levels in Kermanshah province in years 2014–2006.

Figure 2 and Table 1 illustrate that during the study period (2006–14), the suicide attempt rate in females (60.53%) was higher than that in males (39.46%). During the waves, we find that the suicide attempt rate among females and males increased, especially females. Particularly, the suicide attempts in 2012 were higher than in other years. The rate of suicide attempts in males decreased in 2006, 2007, 2008, and 2009, and increased after 2010, while the rate of suicide attempt in females increased during 2006, 2008, 2010, 2012, and 2014. Also, we observed that the suicide attempts increased dramatically in aged 15–64 years. In addition, suicide attempts increased sharply in secondary education from 2006 to 2014.

**Figure 3:**
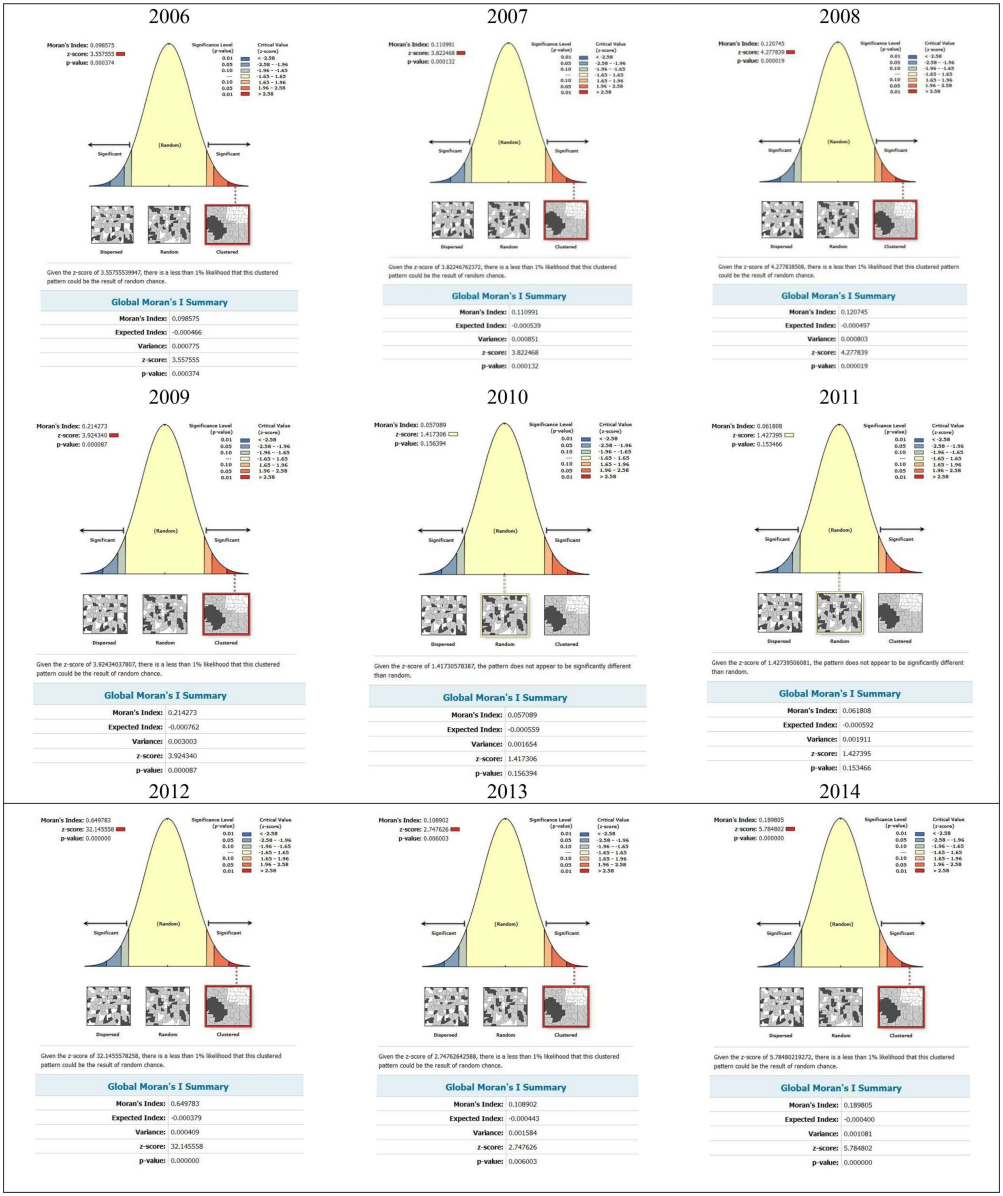
The Global Moran’s I statistic for suicides attempt in the Kermanshah Province, 2006–2014.

The results of the Moran’s I test show that the spatial pattern of suicide attempts in Kermanshah province took a cluster form during 2006–2014. In 2006, the Moran’s I was (Index = 0.09, *z* = 3.55, *p* = *p* < 0.001), but in 2014 the Moran’s I increased (Index = 0.18, *z* = 5.78, *p* = *p* < 0.001), indicating a greater increase in suicide attempt rates in suicide foci Figure 3. This result also indicates a high level of spatial dependence for suicides in Kermanshah province.

**Figure 4:**
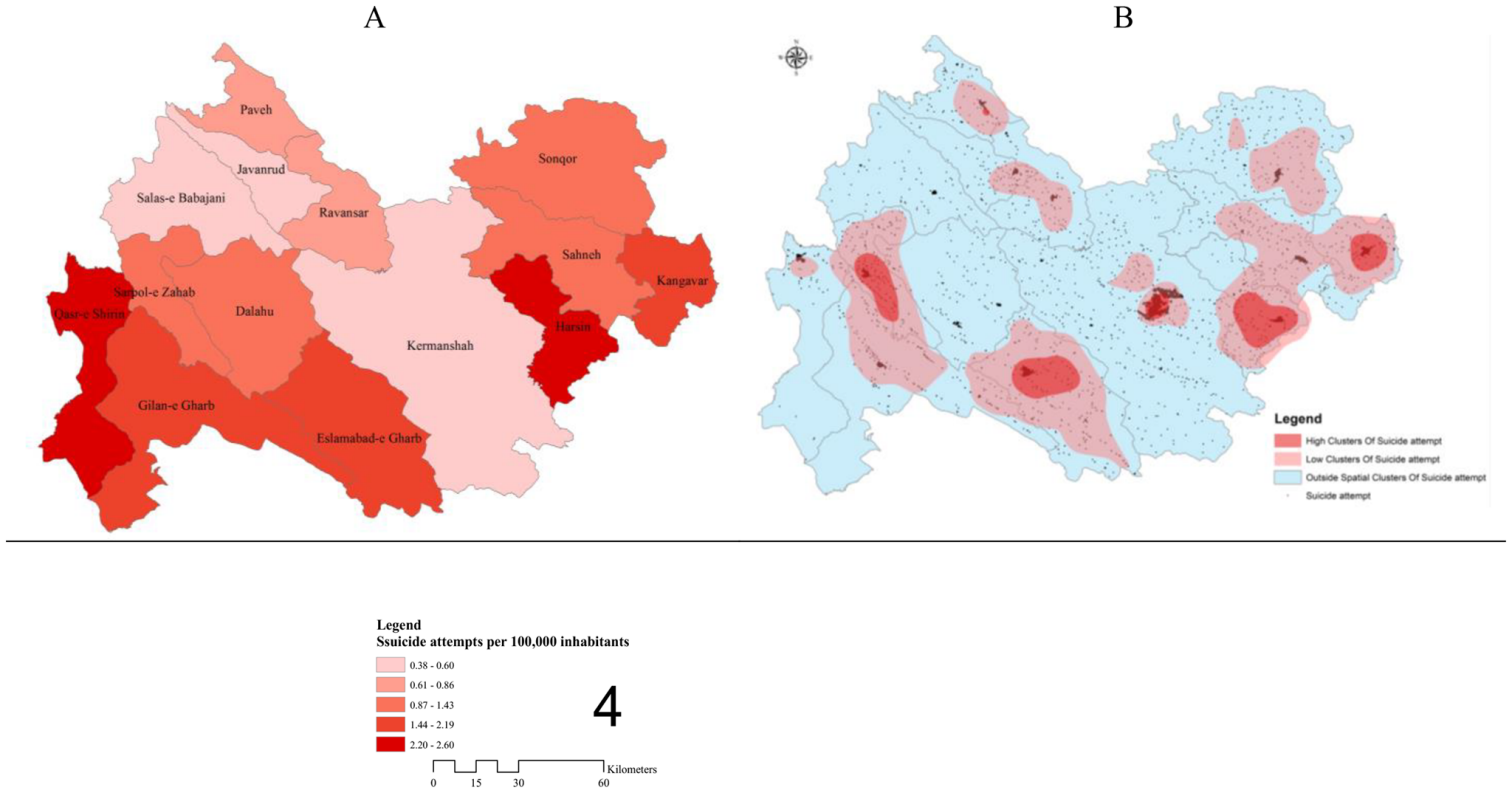
**(A)** Spatial analysis of resident suicide attempts per 100,000 inhabitants. **(B)** Hot spots using KDE in the Kermanshah province, 2006–2014.

Figure 4 and Table 2 present the demographic characteristics of those who attempted suicide within and outside the foci. Accordingly, high clusters and low clusters were identified in Kermanshah province. There were 6 cases of high clusters and 10 cases of low clusters. The results show that suicide attempt in females were (inside foci = 59.72% and out of foci = 67.40%) and in males were (within foci = 40.28% and outside foci = 32.60%).

A chi-square test of independence was performed to examine the relation between males and females (X2 (2, *N* = 18,331) =0.06, *p* < 0.001), between single, marital status, wife’s death, divorced, and widow (X2 (2, *N* = 18,331) =0.02, *p* < 0.048), between educational status (X2 (2, *N* = 18,331) =0.08, *p* < 0.001), and between mean age (X2 (2, *N* = 18,331) =0.11, *p* < 0.001) in within foci and out of foci, and the relation between all the variables was significant, respectively. In addition, the mean age in the foci (*M* = 18.13, SD = 13.58) was higher than that of outside the foci (*M* = 16.99, SD = 14.94).

**Figure 5:**
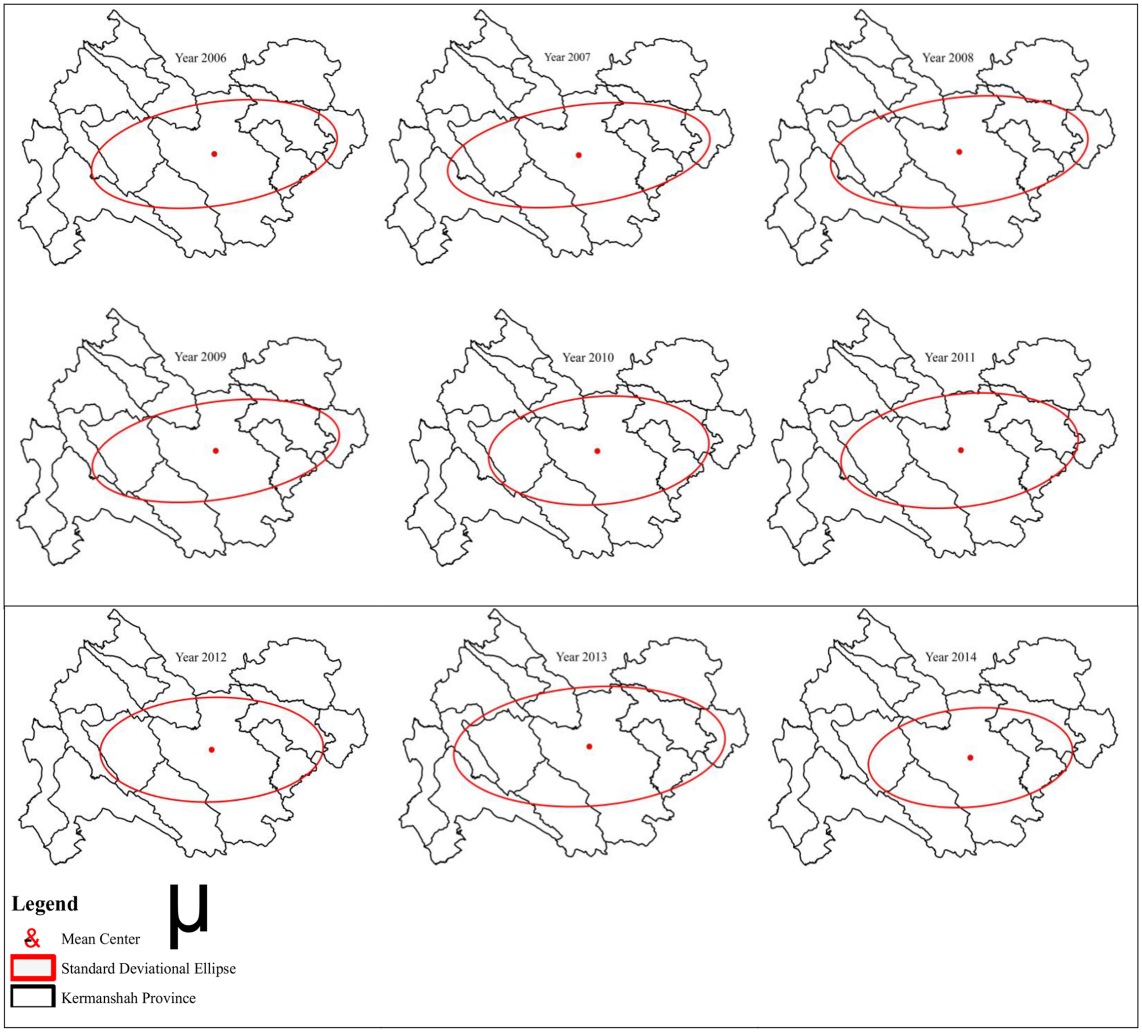
Mean center and SDE suicide attempt in the Kermanshah province during 2006–2014.

The results reveal that during the study period (2006–2014), the mean center for suicide attempts appeared in Kermanshah city. Moreover, the standard deviation of suicide attempts in Kermanshah province shifted from Southwest-Northeast to West–East direction, covering the townships in the Western and Eastern parts of the province; these areas have the highest concentration in the province Figure 5.

## Discussion

Despite previous studies, this study can provide new insights into suicide in Iran. Due to the time of this study, study was not conducted in Iran. Our findings regarding the spatial trend of suicide attempt over a period of time (2006–2014) in the Kermanshah province showed that the center of suicide attempt was located in the Kermanshah city, and the SDE of suicide attempt has shifted from southwest-northeast to west–east with more focus on the Kermanshah city. This situation may have been affected by the increasing migration from other parts of the province to the Kermanshah city. In this regard, the results of studies have shown that poverty, unemployment, inadequate health, and environmental conditions, and also living in high-risk neighborhoods are among the problems faced by migrants (52). These are probably among the factors causing suicide in the Kermanshah province. On the other hand, the results of our study showed that the spatial distribution of suicide attempts in the Kermanshah province was in the clustered form Figure 2. These results were similar to the results of the studies of Fontanella et al. in Ohio, USA (4) as well as Torok et al. studies in Australia. The results of other studies indicate socioeconomic deprivation, low household income, low education, lack of social support have considered as effective factors for suicide cluster formation (53). Likely, the Kermanshah province has faced similar conditions. Therefore, it is suggested to investigate these issues in future studies.

Younger people were at greater risks than older people. Similar results were found in Australia (54). The suicide risk factors among older people are related to employment, depression, bereavement, feelings of social disconnectedness, social exclusion, cognitive impairment and inhibition, illnesses, psychological and physical pain, disability, and neurocognitive and psychological disorders (55, 56). The high suicide attempt rate of young people in Kermanshah may be related to a range of risk factors, such as psychological and economic crisis (unemployment), addiction to drugs as a result of poverty, inappropriate social and economic situation, behaviors, lifestyle, environmental exposure, lack of supervision and community control, weak community structures, and other inherited characteristics (54, 57, 58). A lower average age indicates that more planning is needed to protect and improve young people’s health, especially from a mental health perspective (42, 58). Future research should further investigate the reasons for the lower average age of suicide attempts in the Kermanshah province.

There was a meaningful difference between male and female suicide attempts (*p* < 0.05). Generally, within and outside the foci, females committed suicide more than males. The disparities in sex can be attributed to unequal socio-economic and cultural conditions in Iranian society. Females commit suicide because of inequality in social relationships, traditional societal attitudes toward females, patriarchal culture, forced marriage, polygamy, domestic violence, divorce, and restraints of females in the family (59, 60). In this regard, the result of studies in Kermanshah showed that the suicide rate in this province has been increasing which probably due to unfavorable economic conditions (36, 37, 41–43), as this province has been facing serious problems such as unemployment, poverty and diseases (38, 44–46).

A strong relation between education level and suicide attempts exists both within and outside the foci. Suicidal attempts among people with higher education within the foci were more than that outside the foci. Importantly, the rate of suicide attempts reduced in illiterate people and increased in people with university degrees. Lack of suitable jobs and unemployment may be some of the risk factors for suicide attempts among highly educated individuals (61). More specifically, high unemployment rates negatively affect the morale of educated people and increase suicide attempts (62). This finding is in contrast with existing research, which indicates that the rate of suicide attempt is connected to lower education (63). Low education is closely correlated with suicide attempts and suicidal ideation around the world (64, 65). People with lower education may be at greater risk of a suicide attempt due to insufficient coping skills (58).

The findings of this study show that marriage is associated with a higher risk of suicide attempts. Previous studies on the relation between marriage and suicide attempts have varied results–some argue that marriage and having a family reduces the probability of suicide attempts (59, 66), while others find that suicide attempts are more common in married people (67). In Denmark, at least one indecent of depression occurred in 1,606 male and 1,168 females suicide cases, most of whom were employed and married (68). Studies also argue that compared with unmarried people, married female s and males have a lower suicide risk, while the suicide rate is significantly higher among separated and divorced people than that of never married. These varying results could be due to different cultural patterns in different societies.

### Limitations

This study has several limitations. First, missing data on residential addresses resulted in incomplete geo-coding; however, less than 4% of suicide cases were missing data on residential addresses. Second, the findings might not reflect current high-risk areas as data was from 2006–2014. Third, the socioeconomic factors such as poverty, unemployment, social support and the welfare system were not examined and warrant further research. We recommend that future studies consider them. Ultimately, however, we believe that our measure of spatiotemporal clustering of suicide attempt is an important step toward a richer understanding of suicide attempt in Iran. Due to no research was conducted in Iran to study the spatial distribution of suicide. Our study could be the based studies in future.

## Conclusion

The results of this study show that the spatial pattern of suicide attempts in the Kermanshah were clustered and most hot spots of suicide were formed in and around cities. Clusters of high suicide attempt risk span the rural–urban continuum in Kermanshah province. Younger people compared to older people were at a greater risk. In addition, the rate of suicide attempts decreases in illiterate people and increases in people with university degrees. Being unmarried was associated with a greater risk, while marriage and having a family reduces the probability of a suicide attempt. The findings could help public health practitioners and policymakers prioritise resources and target efforts for suicide attempt prevention, particularly in Iran.

## Data availability statement

The original contributions presented in the study are included in the article/supplementary materials, further inquiries can be directed to the corresponding author.

## Ethics statement

The studies involving human participants were reviewed and approved by the ethics committee and institutional review board of the Medical Association of the Kermanshah University of Medical Science. The patients/participants provided their written informed consent to participate in this study.

## Author contributions

AZa, NK, NF, AZi, RN, SO, RT, KY, and SM were responsible for the study. AZa, NK, and AZi conceptualization and led the paper’s writing. AZa and KY conducted the Literature review and assisted in writing the paper. AZa and NF performed the analysis, assisted in interpreting the data, and writing the paper. RN, SO, SM, and RT assisted with the interpretation of the results and drafting programmatic Implications. AZa and AZi were responsible for the data collection and coordination of the study. AZa co-led the conceptualization, supervised all aspects of writing the paper, and provided extensive comments on the manuscript. All authors contributed to the article and approved the submitted version.

## Funding

The project received financial support from the Deputy Head of the Research & Technology Department of the Kermanshah University of Medical Sciences. The article processing cost is covered by the Kermanshah University of Medical Sciences, Kermanshah, Iran.

## Conflict of interest

The authors declare that the research was conducted in the absence of any commercial or financial relationships that could be construed as a potential conflict of interest.

## Publisher’s note

All claims expressed in this article are solely those of the authors and do not necessarily represent those of their affiliated organizations, or those of the publisher, the editors and the reviewers. Any product that may be evaluated in this article, or claim that may be made by its manufacturer, is not guaranteed or endorsed by the publisher.
